# Delusional Parasitosis in a Patient with Alcohol-induced Psychotic Disorder

**DOI:** 10.7759/cureus.4344

**Published:** 2019-03-29

**Authors:** Blayne Knapp, Emmanuel Tito, Eduardo D Espiridion

**Affiliations:** 1 Family Medicine, West Virginia School of Osteopathic Medicine, Lewisburg, USA; 2 Internal Medicine, West Virginia School Osteopathic Medicine, Lewisburg, USA; 3 Psychiatry, Frederick Memorial Hospital, Frederick, USA

**Keywords:** alcohol induced psychotic disorder, alcoholic hallucinosis, hallucination, delusion, delusional parasitosis, alcohol, psychosis

## Abstract

Alcohol-induced psychotic disorder is a rare complication of chronic alcohol abuse following abrupt alcohol cessation that is characterized by visual, auditory, or tactile hallucinations paired with intact orientation and stable vital signs, distinguishing the condition from delirium tremens and psychotic disorders. The condition, first termed alcoholic hallucinosis, has been described in medical literature for over a century, however, its nosologic classification and psychopathologic characteristics are less well-documented. One such case of alcohol-induced psychotic disorder with multimodal hallucinations of four months duration is described here.

## Introduction

Chronic alcohol abuse may lead to a variety of complications following abrupt alcohol cessation, the mildest and most common presentation being withdrawal symptoms of anxiety, insomnia, tremors, palpitations, and diaphoresis, with the patient’s orientation left intact [1-2]. The more severe presentation of chronic alcohol abuse is that of delirium tremens (DT), aptly named for the delirium, or sudden and severe confusion, that begins two to four days after abstinence. Additional findings of DT include agitation, fever, tachycardia, hypertension, diaphoresis, and hallucinations [3-4]. Less well-characterized is a manifestation of chronic alcohol abuse termed alcoholic hallucinosis, or more recently classified by the International Classification of Diseases-11 (ICD-11) as alcohol-induced psychotic disorder (AIPD), predominantly hallucinatory type [5]. AIPD may manifest with visual, auditory, or tactile hallucinations, delusions of persecution, and mood disturbances, though the level of consciousness and the patient's vital signs remain intact, distinguishing it from DT [6-8]. This condition has been noted in the literature for over a century although the diagnostic characteristics, chronology, and nosologic understanding of the disease are less well-characterized [6-7]. The purpose of this case report is to describe a presentation of AIPD and discuss the clinical assessments that allowed for the differentiation of the psychopathologic characteristics of AIPD from the similarly presenting psychiatric illnesses of schizophrenia, substance-induced psychosis, DT, and organic causes of hallucinosis. Described here is a case of AIPD that manifested in a 55-year-old woman who presented to the community hospital inpatient behavioral health unit with multimodal hallucinations of four months duration.

## Case presentation

A 55-year-old, divorced, multiparous Caucasian female was referred for psychiatric evaluation by her daughter and hospital staff due to her hallucinations and thoughts of suicide. The patient reported a delusion that three men had broken into her home with the intent to harm her and her daughter. Upon the daughter’s realization of the delusion, the patient was brought to the community hospital for assessment.

Upon psychiatric evaluation, the patient reported her hallucinations began four months prior as auditory hallucinations of a young girl and a rodent co-inhabiting the patient’s house. The patient also described gradually intensifying visual and tactile hallucinations of scabies crawling out of her skin, referred to as delusional parasitosis. The patient presented with self-inflicted excoriation and burn injuries to her extremities, face, and abdomen that were sustained in her efforts to rid herself of the parasitic delusions. The patient resorted to picking at her skin, applying bleach and ammonium-based cleaning products and scabicidal agents to her skin while enduring these delusions. The patient had previously claimed to have proof of parasitic specimens collected from her body that she had visualized under a magnifying glass, though she reported several dermatologists were unable to confirm her findings. The patient claimed that, eventually, she was able to communicate with the parasitic delusions, as she was unable to get rid of them and claimed the scabies eventually acquired personalities that she would talk to.

The event that led to the patient’s presentation for psychiatric evaluation involved persecutory delusions, parasitic delusions, and intrusive thoughts that she should swallow objects, though she reported she was able to dissuade herself from doing so. The patient endorsed poor concentration, memory loss over the past six months, as well as occasional feelings of helplessness, anhedonia, and insomnia. The patient admitted to a longstanding history of alcohol abuse, recent relapse, and noncompliance with her naltrexone prescription. Her last report of alcohol consumption was two days prior to admission wherein she consumed one bottle of plum wine over a span of two days.

The patient attributed the alcohol relapse and current disturbances to recent life stressors. She had assumed the role of caregiver for her mother who suffered from advanced dementia and had recently passed away. She also had increased interactions with her ex-husband whom the patient claimed had been physically and psychologically abusive towards her during their marriage. The patient voiced concern over the possibility of her current symptoms being related to a neuropathologic process such as dementia or Parkinson’s disease. She complained of a recent onset of fine tremor in her hands and intermittent ataxia of four months duration, for which she expressed interest in being evaluated by a neurologist during treatment.

In addition to these life stressors and history of alcohol abuse, the patient had a history of bipolar disorder, depression, attention deficit hyperactivity disorder (ADHD), and post-traumatic stress disorder from past sexual abuse sustained when she was a teenager. The patient denied any history of illicit substance abuse other than alcohol. The patient's non-psychiatric medical illness history was significant for hypertension and tachycardia. She reported past hospitalizations for the birth of her two children, both of which were vaginal, to term, and uncomplicated. She reported no past surgical history and no allergies. The patient's family history was significant for alcohol use disorder and cognitive impairment related to dementia. There was no evidence of psychiatric illness, including delirium, psychosis, mood disorders, or suicide in family history. The patient reported her alcohol use began at age 15 following an attack in which she was raped, after which she suffered from post-traumatic stress disorder and alcohol abuse. Her alcohol use steadily intensified in 2003 when her husband became increasingly abusive towards her. The patient reported consuming an of average one gallon of vodka each day until her first rehabilitation effort occurred in 2011. She denied irritability, anxiety, tremors, confusion, or seizures when abstaining from alcohol use at that time. She denied delusions and hallucinations when abstaining from alcohol in the past. She denied a history of suicidal behavior, drug abuse, or prior hospitalizations for alcohol intoxication. The patient was enthusiastic about her recovery efforts but reported her recent relapse as occupational stresses mounted and her mother’s health waned. Her alcohol abuse eventually cost the patient her job in 2014, so she assumed care of her mother who was then suffering from advanced dementia. At this time, the patient began seeing a community psychiatrist and alcohol rehabilitation treatments commenced for the second time. Under this provider’s care, the patient’s underlying psychiatric illnesses including bipolar disorder, depression, anxiety, and ADHD were also addressed. With the help of her family members, the patient successfully completed an intensive alcohol detoxification program before relapsing in 2015 following her mother’s death. The patient stated her alcohol use has contributed to her ongoing unemployment and recent tensions in her family.

The patient reported past non-compliance with her Adderall (mixed amphetamine salts) prescription dose of 30 mg per day and bupropion prescription of 450 mg per day for ADHD and depression, respectively. She indicated that occasionally, she would take twice her daily dose of Adderall and reported that recently she had been taking this medication as prescribed until three days prior to her admission for psychiatric evaluation.

**Figure 1:**
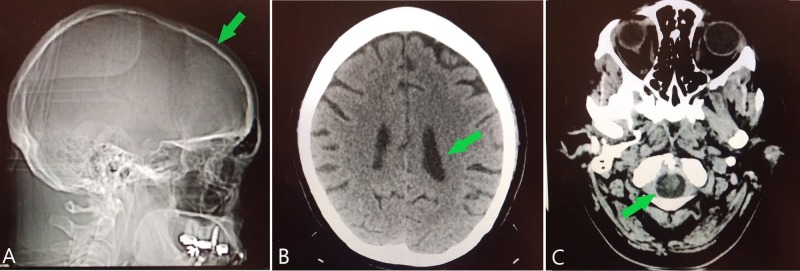
Illustrative imaging of the head Saggital X-ray (A) demonstrates intact skull line (arrow), indicating no gross deformity or sign of trauma. Axial non-contrast computed tomography of the head (B) indicates appropriate size of lateral ventricles (arrow), no midline shift, or masses with appropriate parenchymal symmetry, and (C) demonstrates no pathology or space-occupying lesions in the brainstem or structures within the foramen magnum (arrow). These findings make an organic or pathologic cause of psychosis less likely.

The patient's physical exam was within normal limits with pertinent findings being the excoriated lesions along the patient’s shins, arms, and abdomen. A comprehensive mental status examination was found to be appropriate and her vital signs were normal, except for an elevated blood pressure that was addressed and controlled with hydrochlorothiazide 12.5 mg per day and metoprolol 100 mg twice per day. Her urinalysis and complete blood count results were within normal limits. The patient's comprehensive metabolic panel was significant for elevated aspartate aminotransferase (AST) and alanine aminotransferase (ALT). The patient's urine toxicology screen was positive for amphetamines and negative for cocaine, tetrahydrocannabinol (THC), phencyclidine (PCP), and other psychoactive substances. Her urine ethyl alcohol levels were less than 10 mg/dL. X-ray and non-contrast computed tomography scan imaging studies were ordered to rule out organic causes of hallucinosis or intracranial pathology and the results were unremarkable (Figure 1). Outcomes of the patient's comprehensive neurological testing were similarly unremarkable. The patient's psychiatric evaluation revealed no evidence of psychomotor agitation, pressured speech, her mood was anxious, and affect was appropriate. There were no referential or paranoid ideations or loose associations. The patient denied any present thoughts of broadcasting, insertion, or withdrawal. She admitted to mild and infrequent visual and tactile hallucinations of bugs crawling out of her skin but stated that, at present, she had accepted that these hallucinations and the delusions of intruders in her home had not been real. She was unable to perform serial sevens or spelling words forward and backward on psychometric testing. The patient displayed poor insight, judgment, and impulse control during the examination.

Treatment of the patient’s skin lesions included cleaning with normal saline and silver sulfadiazine cream. Antipruritic antihistamine cream and topical mupirocin antibiotics were applied to the patient's skin.

The patient’s psychotic symptoms were addressed with 5 mg olanzapine daily that was administered at bedtime. The dosing of the patient’s 450 mg of bupropion was tapered and restarting the Adderall prescription was deferred. The reason for tapering the bupropion and deferring re-prescribing Adderall was because both medications have the potential to induce acute mania as a side effect. Psycho-education was provided to the patient regarding her currently prescribed medications and each potential adverse effect. The patient expressed strong motivation for future abstinence from alcohol and enthusiastically agreed to receive an extended-release injectable form of naltrexone prior to her discharge.

## Discussion

AIPD is known to manifest in the presence of withdrawal of chronic alcohol consumption, though it may occur in the setting of intoxication as well [6-7]. Hallucinations may be part of a primary psychotic disorder or secondary to substance abuse, including cocaine, alcohol, and amphetamines, the latter which the patient tested positive for on urine toxicology screening [8]. The Diagnostic and Statistical Manual of Mental Disorders, Fifth Edition (DSM-5) states that there must be evidence of significant hallucinations or delusions in considering a diagnosis of substance-induced psychotic disorder [9-12]. DSM-5 outlines four additional factors to meet the diagnosis. First, hallucinations or delusions that start during or soon after intoxication or withdrawal of the substance in use is definitively known to cause AIPD [12]. The patient described in this case presentation experienced visual, tactile, and auditory hallucinations that occurred regularly for four months duration although the report of her alcohol use was irregular during this time. The patient’s chronic history of alcohol abuse paired with her recent relapse and pattern of irregular consumption suggest the hallucinations and delusions were tied to this period of alcohol use. Second, the patient’s symptoms were not better explained by another psychotic disorder unrelated to the substance [10-13]. A diagnosis of schizophrenia was excluded as she was lacking in negative symptoms, disorganized thoughts, and behavior. The patient never met criteria for the diagnosis for bipolar disorder with psychotic features as symptoms of mania were absent and disorganized thoughts and speech patterns were also absent. Third, the symptoms of psychosis were found to be present in the absence of delirium, as the patient's comprehensive mental status examination showed no evidence of fluctuating levels of consciousness [12]. She adequately participated in the interview and displayed no sign of disorientation or mood swing. Fourth, the symptoms the patient was experiencing were significantly interfering with daily living activities, such as work, where her chronic alcohol use led to her dismissal from the most recent employer and had contributed to relational tensions with family members [12].

It is documented that patients with AIPD may experience delusional parasitosis occurring in the setting of chronic alcohol abuse, which the patient described in detail [6,11]. She reported the tactile sensation of feeling bugs crawling on her skin, which led her to excoriate and apply cleaning chemicals to her skin in an effort to rid herself of the delusions. Identifiable skin lesions were examined and infestation was ruled out by wound care evaluation and treatment prior to her psychiatric evaluation.

Given the patient’s normal vital signs and normal mentation, she lacked physical exam criteria that would rule in a diagnosis of DT [3-4]. The history of alcohol use disorder and her presenting psychiatric symptoms of hallucinations and delusions ruled out a diagnosis of uncomplicated substance abuse [13-14]. The portions of her past medical history and current medication regimen that disabled definitively ruling out substance-induced psychosis were the prescriptions for Adderall and Wellbutrin, two medications that are known to contribute to or possibly induce psychosis. AIPD was a more likely diagnosis, as the patient's present illness overlapped with her recent alcohol relapse, whereas the two possible offending medications had been prescribed for several years [11,15]. The patient’s CT scan failed to demonstrate space-occupying lesions or other abnormal findings, making an organic cause of psychosis less likely [11,16]. Normal findings on neurologic exam made it highly unlikely that her presentation was due to ischemic brain injury, and, for this reason, magnetic resonance imaging (MRI) of the head was not performed. Given that the patient met the four DSM-5 criteria for AIPD, alcohol-induced psychosis was a more likely diagnosis, making discontinuation of the substance use an essential part of her treatment [7-8,12,16].

Appropriate recognition of AIPD is paramount and will enable appropriate treatment of the disorder. Discontinuation of alcohol use is imperative to treat this condition on an individual basis and on a population-based level of care [16-17]. There is mounting evidence that if AIPD is left untreated, it may lead to prolonged schizophrenia-like symptoms, delirium, and increased risk for premature death, which is why further research must be done to define the nosologic characteristics of AIPD, to determine recommended adjunct treatments, and determine the prognosis of the disorder [7,17-18].

## Conclusions

The patient described in this case study fits the criteria for AIPD given her alcohol use history, auditory hallucinations, parasitic delusions, persecutory delusions, as well as her reports and physical evidence of delusional parasitosis. It is difficult to distinguish the timeline of AIPD in how it relates to the patient’s history of chronic alcohol abuse and her report of the psychiatric disturbances that occurred for a span of four months. Though more research must be done to definitively define the intricacies of this patient’s psychiatric history, social stressors, chronic substance abuse, and possible iatrogenic contributions to the psychosis she experienced, her condition was appropriately managed. She was treated and cleared by medical and psychiatric management. Psychoeducation and medication management were also crucial in addressing this patient's substance use disorder to prevent her from future episodes of AIPD.
